# Fungi of Great Salt Lake, Utah, USA: a spatial survey

**DOI:** 10.3389/ffunb.2024.1438347

**Published:** 2024-09-13

**Authors:** David L. Parrott, Bonnie K. Baxter

**Affiliations:** Great Salt Lake Institute, Westminster University, Salt Lake City, UT, United States

**Keywords:** Great Salt Lake, hypersaline adaptation, halophilic fungi, eDNA, microbial community diversity

## Abstract

The natural system at Great Salt Lake, Utah, USA was augmented by the construction of a rock-filled railroad causeway in 1960, creating two lakes at one site. The north arm is sequestered from the mountain snowmelt inputs and thus became saturated with salts (250-340 g/L). The south arm is a flourishing ecosystem with moderate salinity (90-190 g/L) and a significant body of water for ten million birds on the avian flyways of the western US who engorge themselves on the large biomass of brine flies and shrimp. The sediments around the lake shores include calcium carbonate oolitic sand and clay, and further away from the saltwater margins, a zone with less saline soil. Here a small number of plants can thrive, including *Salicornia* and *Sueda* species. At the north arm at Rozel Point, halite crystals precipitate in the salt-saturated lake water, calcium sulfate precipitates to form gypsum crystals embedded in the clay, and high molecular weight asphalt seeps from the ground. It is an ecosystem with gradients and extremes, and fungi are up to the challenge. We have collected data on Great Salt Lake fungi from a variety of studies and present them here in a spatial survey. Combining knowledge of cultivation studies as well as environmental DNA work, we discuss the genera prevalent in and around this unique ecosystem. A wide diversity of taxa were found in multiple microniches of the lake, suggesting significant roles for these genera: *Acremonium, Alternaria*, *Aspergillus*, *Cladosporium*, *Clydae*, *Coniochaeta*, *Cryptococcus*, *Malassezia*, *Nectria*, *Penicillium*, *Powellomyces*, *Rhizophlyctis*, and *Wallemia*. Considering the species present and the features of Great Salt Lake as a terminal basin, we discuss of the possible roles of the fungi. These include not only nutrient cycling, toxin mediation, and predation for the ecosystem, but also roles that would enable other life to thrive in the water and on the shore. Many genera that we discovered may help other organisms in alleviating salinity stress, promoting growth, or affording protection from dehydration. The diverse taxa of Great Salt Lake fungi provide important benefits for the ecosystem.

## 1 The Great Salt Lake ecosystem

An enormous and iconic terminal lake, Great Salt Lake (Utah, USA), is a significant body of water on the Pacific Flyway, feeding ten million birds annually (Sorensen et al., 2020). The ecology is dependent on two invertebrates in the open water, the brine shrimp and brine fly, and a large expanse of wetlands for nesting and feeding (Baxter, 2024). But this incredible ecosystem is only in the south arm of the lake, which receives water from the snowpack of the nearby Wasatch mountains. Around 1960, construction of a causeway for rail transport disrupted the lake; the north arm of Great Salt Lake was segmented away from the mountain runoff, which caused this part of the lake to become saturated (Adams, 1964). Since salinity is a driver of biological complexity, the microbial community of the north arm (250-340 g/L salts) is distinct from the south arm (90-190 g/L) (Baxter and Zalar, 2019). The thalassohaline water chemistry mimics the ion ratios of the oceans, largely sodium and chloride with a high sulfate concentration (Saccò et al., 2021).

Great Salt Lake has been shrinking as water diversions for consumptive uses have increased (Null and Wurtsbaugh, 2020), a fate similar to other terminal lakes globally (Wurtsbaugh et al., 2017). Climate change pressures have reduced inflows, exacerbating the water loss (Wang et al., 2018; Meng, 2019; Baxter and Butler, 2020; Hassan et al., 2023). This has altered the microbial microniches at the lake, changing salinity and exposing new environments such as tar seeps (Kornhauser et al., 2020) and a broad expanse of encroaching shoreline plants (Bradbury and Parrott, 2020). In addition, evaporite minerals, including halite (NaCl) and gypsum (CaSO_4_·2H_2_O), are deposited as the water recedes (Eardley and Stringham, 1952; Rupke and McDonald, 2012).

Thus, investigations of Great Salt Lake microbial communities should consider the many facets of the ecosystem, including the water, minerals, sediment, petroleum seeps, soils, shoreline plants, salinity gradient, and invertebrate flora. The baseline hurdle is hypersalinity, but the microorganisms that thrive here are poly-extremophiles as they must also deal with challenging chemistry. The prokaryotes of Great Salt Lake, especially the archaea, have been probed for ability to deal with desiccation, and radiation resistance (Bayles et al., 2021), but few studies until recently have focused on the fungi. We are amid several projects at the lake; this manuscript is a compilation of our findings, and that of others, with respect to fungi data from the Great Salt Lake ecosystem to date.

## 2 Cultivation of fungi at Great Salt Lake

Fungi have prolific dispersal mechanisms (Chaudhary et al., 2022) and their extremophile repertoire in hypersaline systems is unmatched (Gostinčar and Gunde-Cimerman, 2023), yet microbial explorations at Great Salt Lake have mostly ignored the possibility of fungi in the ecosystem. The first reported cultivation of a fungus from the lake was regarding a *Cladosporium* species from a submerged piece of wood floating in the brine (Cronin and Post, 1977). A yeast isolation study in the south arm resulted in culturing *Debariomyces hansenii* and *Metschnikowia bicuspidata*, the latter possibly associated with brine shrimp (Butinar et al., 2005). A recent, more systematic cultivation effort isolated 32 strains from 11 genera (Baxter and Zalar, 2019). This study cultured species in the brine of both the north and south arms. In addition, the authors cultivated fungi from calcium carbonate oolitic sand, halite crystals, and the Rozel tar seeps. Drawn from all these sources, known cultivars are assembled into **Table 1**. All cultivation work except for the first isolated *Cladosporium* used isolation methods from Gunde-Cimerman et al. (2000) and others. Four different procedures were employed including plating on high salt media after filtration, deploying agar baits in tubing in the brine, and enriching species with additives such as yeast extract and glucose.

**Table 1:** Cultivars of Fungal strains at Great Salt Lake arranged alphabetically under phylum. Sampling locations, source, and references are included.

Strain	Site/Source^Ref^
*Acremonium* sp.	NA/Halite^1^
*Acremonium egyptiacum*	SA/Brine^1^
*Alternaria arborescens*	NA/Ooids^1^
*Alternaria arborescens*	NA/Halite^1^
*Alternaria multiformis*	NA/Brine^1^
*Alternaria rosae*	NA/Brine^1^
*Aspergillus flavus*	SA/Brine^1^
*Aspergillus fumigatus*	NA/Tar seeps^1^
*Aspergillus fumigatus*	SA/Brine^1^
*Cladosporium* sp.	SA/Brine-soaked wood^2^
*Cladosporium cladosporioides*	SA/Brine^1^
*Cladosporium halotolerans*	SA/Brine^1^
*Cladosporium cladosporioides*	NA/Ooids^1^
*Cladosporium cladosporioides*	NA/Brine^1^
*Cladosporium pseudocladosporioides*	SA/Brine^1^
*Cladosporium herbarum*	NA/Brine^1^
*Coniochaeta polymorpha*	NA/Rozel tar seeps^1^
*Debaryomyces hansenii*	SA/Brine^3^
*Debaryomyces hansenii*	SA/Brine^1^
*Metschnikowia bicuspidata*	SA/Brine^3^
*Nectria punicea*	NA/Brine^1^
*Neocamarosporium* sp.	NA/Ooids^1^
*Parengyodontium album*	NA/Brine^1^
*Penicillium* aff. *mononematosum*	NA/Ooids^1^
*Penicillium buchwaldii*	NA/Brine^1^
*Penicillium crustosum*	SA/Brine^1^
*Stemphylium* sp.	NA/Halite^1^
*Wallemia muriae*	NA/Brine^1^
*Wallemia sebi*	SA/Brine^1^

NA, north arm of Great Salt Lake; SA south arm of Great Salt Lake. Reference footnotes in the table are defined as follows: (Baxter and Zalar, 2019)^1^, (Cronin and Post, 1977)^2^, (Butinar et al., 2005)^3^.

## 3 Environmental DNA studies of fungi at Great Salt Lake

The “fungi gap” is not limited to cultivation investigations; molecular studies of many systems are only beginning to accrue mycology data (e.g. Zhang et al., 2015). In environmental DNA (eDNA) work, it is not uncommon for fungal taxa to be missing in the analysis. This could be due to the resistance of some fungi to DNA extraction due to their thick cell walls (Gostinčar et al., 2014). Alternatively, it could be because fungi are more difficult to type with simple probes than prokaryotes owing to limited variability in target genes and the dearth of available genome sequences in databases (Ruiz and Radwan, 2021). Using molecular techniques has helped assess the mycological diversity at Great Salt Lake, even with these limitations. In our work, we employed bead-beating DNA extraction kits to better homogenize the cellular material (Tedersoo et al., 2022) and used replicate extractions to avoid overwhelming the spin column matrix (Dickie et al., 2018). Both of these strategies have been shown to maximize the resulting DNA quantity.

**Figure 1:**
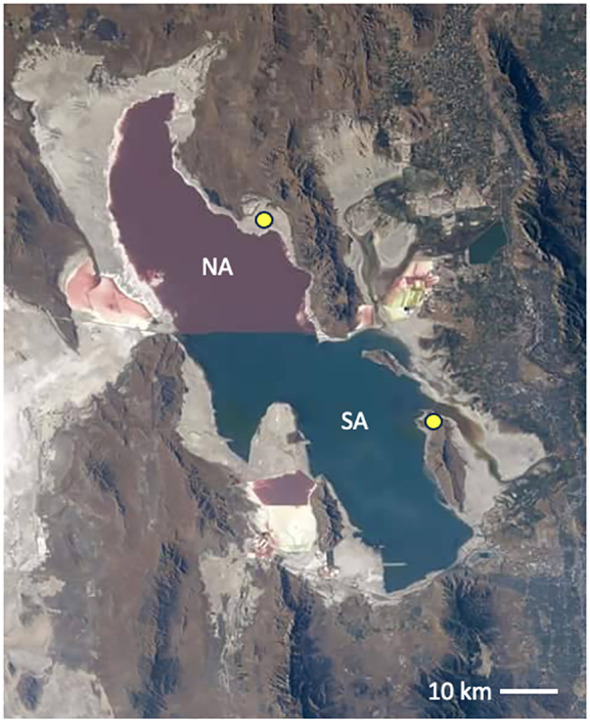
Great Salt Lake as imaged from the International Space Station 2022. Image credit: NASA, public domain. The isolated north arm (NA), above the railroad causeway, is hypersaline and pink in color from the haloarchaea enrichment (Baxter and Zalar, 2019). The south arm (SA) is tinted green from the phytoplankton prevalent in the less saline water. Yellow circles indicate the shore sampling locations for the work detailed here in both the NA and SA.

In several ongoing projects, we have explored the north arm lake water and sediment, gypsum crystals that form in the clay, petroleum seeps, the north shoreline soils, and the plant rhizosphere microbiomes along the margins. We have also sampled the south arm microbialite mats in the benthic zone, the *Ephydra gracilis* (brine fly) pupae attached to the mats, the south shoreline soils, and the plant rhizosphere microbiomes in this area. The location of the (north and south) shore samples is marked with yellow circles on the image in **Figure 1**.

We sampled from this variety of locations and microniches around Great Salt Lake. Samples from the north arm were taken at Rozel Point (N 41.43783°, W−112.67103°), and the south arm samples were collected from Antelope Island State Park (N 41.0639°, W −112.2487°) (**Figure 1**). The soil sampling was done seasonally and with replicates to represent a large area. For each soil sample type and season, three replicates were taken and pooled. Sampling occurred once per season (June-August) at each location for four seasons (2019-2022). DNA was isolated from each of the soil samples using MP Bio FastDNA Spin Kit for Soil (MP Biomedicals, Santa Ana, CA, USA). Petroleum samples were taken from three individual seeps at the north arm Rozel tar seeps and frozen. These were ground with a sterile ceramic mortar and pestle into a fine soil-like consistency, then extracted using the same kit.

Other samples required a different treatment for extraction. Microbialites from two locations, brine fly pupae, and surface-sterilized gypsum were crushed in separate experiments then sterile molecular grade water was added to make a slurry. In each case, the supernatant was filtered through a 0.45 µm Pall MicroFunnel Filter (Show Low, AZ, USA). The north arm brine (Winogradsky column) samples were also filtered in this way. In each separate sample, the DNA was extracted from the membrane of the filter using the DNeasy PowerWater Kit (Hilden, Germany).

**Figure 2:**
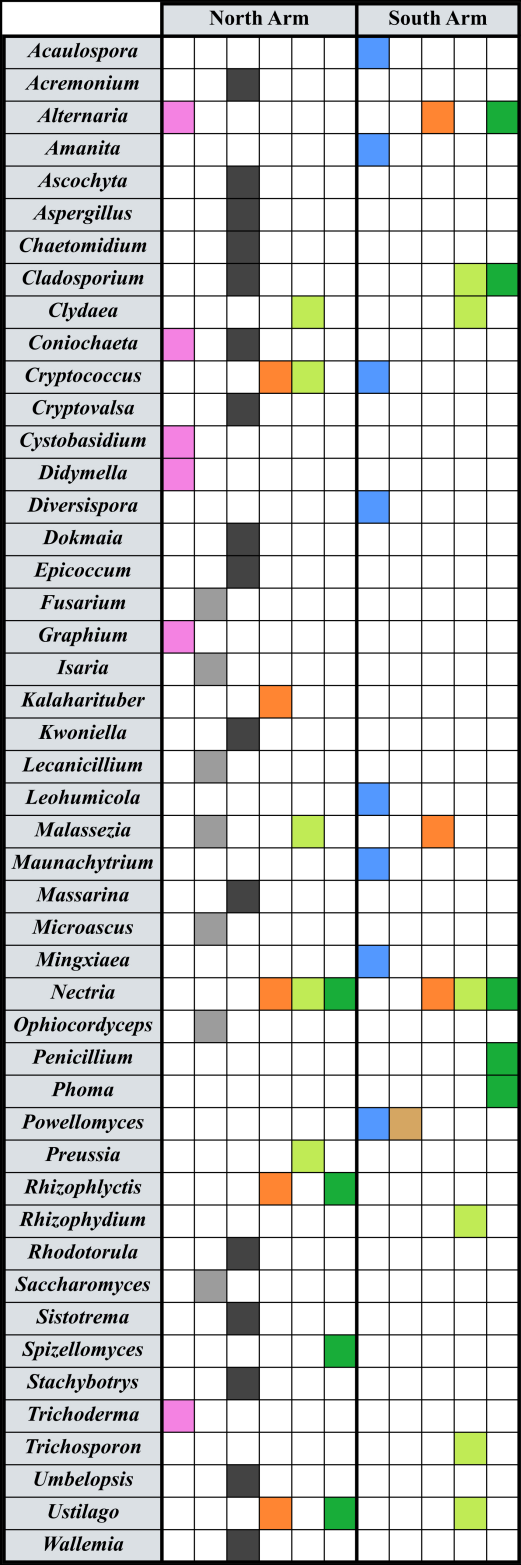
Fungal taxa detected in eDNA work at Great Salt Lake. Genera are listed in the left column, and filled boxes indicate amplification of ITS variable regions. North arm sites are on the left of the graphic, and south arm on the right. Colors indicate the presence of that genus in a certain location: Pink represents taxa detected in a north arm water and sediment Winogradsky column (De Leon et al., 2023); gray, gypsum crystals; black, tar seeps at Rozel Point; blue, microbialites; tan, brine fly pupae; orange, shoreline soils; light green, *Salicornia* spp. roots; dark green, *Suaeda* spp. roots.

Isolated total genomic DNA from all sources was sent out to assess fungal diversity with PCR and DNA sequencing (www.mrdnalab.com, Shallowater, TX, USA). The genomic DNA was amplified with primers for fungal internal transcribed spacers (ITS) 1 and 2 using “ITS1-2” forward and reverse primers (Walters et al., 2016; White et al., 1990). Bacterial tag-encoded FLX amplicon pyrosequencing (bTEFAP) long read 5k ITS1-4 diversity assay was used for the identification of fungi (Dowd et al., 2008). MRDNA provided the abundance measures for each Operational Taxonomic Unit (OTU) represented. Sequencing controls allowed us to delete any contaminating background DNA contributed by the elution buffer. Final OTUs were taxonomically classified using BLASTn against a curated database derived from NCBI (www.ncbi.nlm.nih.gov) and compiled into each taxonomic level by abundance. In the analysis, the data were binned at the level of Genera. Casting this wide net, we have detected a diversity of fungal genera in the sampled microniches (**Figure 2**). All of our data sets were pruned to include only genera at 4% or above in abundance at that site, which excluded those taxa present in insignificant numbers in our datasets. However, the files uploaded to Genbank contain all the sequences (see Data Availability Statement).

## 4 Analysis of taxa from a spatial perspective

When analyzing data for spatial insights, it is important to acknowledge the nimble dispersal mechanisms of fungi, especially at a location like Great Salt Lake with significant wind, water, and dust (Golan and Pringle, 2017; Chaudhary et al., 2022). Some of our data highlight fungal species that are likely introduced to the sampled location versus endemic at that site. For example, gypsum crystals (**Figure 2**), which are embedded in the barren salt playa, appear to have entombed fungi (or their spores) that are associated with insects and plants. *Saccharomyces bayanus* (Naumov et al., 2011), *Microascus cirrosus* (Kumar et al., 2023), and *Fusarium brachygibbosum* (Tirado-Ramirez et al., 2021) are all found on plants; *Ophiocordyceps sinensis* infects caterpillars (Zhang et al., 2012) and *Isaria fumosorosea* causes disease in a variety of insects (D’Alessandro et al., 2011). *Malassezia cuniculi* has been found on animal skin (Gaitanis et al., 2012), and *Lecanicillium fungicola* is a mushroom pathogen (Banks et al., 2019). There are no insects or plants living in this area of the dried hypersaline lakebed. We may be capturing genetic signals of fungi thriving in the surrounding sagebrush steppe, and not just at the precise site sampled.

Among the cultivars (**Table 1**) and the amplified signatures from eDNA studies (**Figure 2**), some genera emerge as significant or prevalent. These interesting stories give the data more meaning and may suggest the roles of fungi across the various microniches of the ecosystem, or as indicated above, they may indicate the breadth of dispersal. The following discussion examines genera that are prevalent in more than one location at Great Salt Lake, listed in alphabetical order. In the eDNA experiments, we have listed the percent abundance for the associated amplicons for that particular dataset.

We noted evidence for *Acremonium* species around various sites at Great Salt Lake. *A. psammosporum* sequences were amplified from DNA extracted from the Rozel tar seeps on the north shore (4.4% abundance) (**Figure 2**) and another species was cultured near that site from halite crystals (**Table 1**). A species associated with humans and animals, *A. egyptiacum* (Summerbell et al., 2018), was grown from samples of south arm brine (**Table 1**). *Acremonium* species are endemic to marine habitats (Jones et al., 2009), and their presence at Great Salt Lake is consistent with these marine-borne fungi in other studied thalassohaline lakes that were once connected to the oceans (Grum-Grzhimaylo et al., 2018).

Species in the genus *Alternaria* are known for mitigating salt and nutrient stress in plants (Tiwari et al., 2023). Therefore, finding *Alternaria embellisia phragmospora* amplicons associated with south arm soil (27.5%) and *Suaeda* plant roots (4.2%) (**Figure 2**) should not be surprising. However, a number of *Alternaria* species are found at the Great Salt Lake north arm site not alongside plants. In the Winogradsky column from the brine and sediment, *A. alternata* was detected (9%) (**Figure 2**). Other *Alternaria* species near the north shore included *A. arborescens* cultured from minerals (both halite crystals and calcium carbonate oolitic sand), and both *A. multiformis* and *A. rosae* isolated from north arm brine (**Table 1**).

In many ecosystems, including those designated as extreme, *Aspergillus* species are a common component of the soil microbiota, serving beneficial roles in addition to saprophytic ones (Nayak et al., 2020). This genus is noted for its ability to solubilize phosphates, recycle carbon and nitrogen, and decontaminate soils. In the south arm, *A. flavus* and *A. fumigatus* were cultured from brine (**Table 1**). In the north arm, *A. niger* eDNA was detected at the Rozel tar seeps (17%) (**Figure 2**) and *A. fumigatus* was cultured from these petroleum seeps (**Table 1**). These species have been associated with other sites rich in hydrocarbons (Ai-Jawhari, 2014).

The first fungus ever reported at Great Salt Lake was a *Cladosporium* species, cultivated from wood that was found floating in the water (Cronin and Post, 1977). Subsequent studies isolated *C. cladosporioides, C. halotolerans*, and *C. pseudocladosporioides* from the south arm lake brine, and *C. cladosporioides* and *C. herbarum* from north arm brine (**Table 1**). *C. cladosporioides* was grown from ooids on the north arm shore (**Table 1**), and this species’ sequences were also amplified from the Rozel tar seeps (6.5%) (**Figure 2**). Our eDNA studies presented here show evidence of *C. davidiella tassiana* in the root microbiome of both plant species tested, *Salicornia* from the north arm (17%) and *Suaeda* from the south arm (6.7%) (**Figure 2**). Since the members of this genus have demonstrated abilities to protect plants against cellular stressors (Răut et al., 2021), the *Cladosporium* associated with the lake water, tar, or minerals may be due to effective dispersal versus a role in those microniches.

*Clydae vesicula* was observed in root microbiomes of *Salicornia* on both shorelines (17% north; 4.7% south) (**Figure 2**). Such Chytridiomycota in freshwater systems are typically parasites of zooplankton that use motile zoospores in their aquatic lifestyle (Kagami et al., 2014). In marine systems they have been observed inside phytoplankton (Gleason et al., 2015; Comeau et al., 2016), even diatoms in Arctic Sea ice (Hassett et al., 2017). *C. vesicula* was recently found in root microbiomes of rice plants in rainfed soils, which are likely wet enough for zoospore movement (Barro et al., 2022); perhaps the spring runoff of snowmelt that brings water to the Great Salt Lake ecosystem can also allow these chytrids to flourish in the *Salicornia* rootzone.

The genus *Coniochaeta* is implicated in other arid Utah biomes in carbon and nitrogen cycling (Challacombe et al., 2019), but it is best known for its association with wood necrosis in plants (Damm et al., 2010). We observed signatures of *C. lecythophora* in the north arm Winogradsky column (9.2%) (**Figure 2**, and De Leon et al., 2023) suggesting it resides in that part of Great Salt Lake or was introduced from the landscape. In the north shore tar seeps, this species was also detected by eDNA (5.3%) (**Figure 2**), and *C. polymorpha* was cultivated from the tar (**Table 1**). There does not appear to be evidence for *Coniochaeta* thriving in hydrocarbon-rich habitats, underscoring the possibility of these strains being blown in from the nearby arid landscape.

The genus *Cryptococcus* includes both yeasts and filamentous species and is considered cosmopolitan in its distribution (Zajc et al., 2014). Two species of Cryptococcus, *C. diffluens and C. saitoi*, were identified in our study (**Figure 2**), although their function remains unknown. Both species were present in soil samples and in the rhizosphere of *Salicornia* from the north arm sampling location (11.5%), which has highly elevated soil salinity levels (Bradbury and Parrott, 2020). The species are both encapsulated yeasts, which have been shown to be salt tolerant (Zajc et al., 2014).

*Malassezia* yeasts are typically animal skin mycobiota, which are usually commensal but can become pathogenic (Torres et al., 2020). Meuser et al., 2013 detected *Malassezia* sp. in the Great Salt Lake south arm water column via 18S rRNA primers (≤ 3.6%) (Meuser et al., 2013). The work presented here (**Figure 2**) indicates presence of this genus within gypsum crystals (*M. cuniculi*) and *Salicornia* root microbiomes (*M. restricta*) at the north arm lake shore and associated with the soil samples from the south arm (*M. globosa*). We were concerned about the possibility of these species being introduced from human hands due to their documented relationship with mammals. However, they grow well in invertebrate model systems (Torres et al., 2020) and have been found in nematodes (e.g. Renker et al., 2003). A recent discovery of nematodes in the south arm lake water suggests the *Malassezia* could indeed have an animal host in the lake and perhaps in the shore sediment (Jung et al., 2024).

*Nectria*, a genus of saprophytic ascomycete fungi associated with decaying wood, plays a role in the breakdown of plant material (Chung et al., 2019). *Nectria mauritiicola* DNA was detected in both soil samples (north 35%; south 33.3%) as well a high association with all plant roots at both sampling sites [north arm *Salicornia* (15.6%), *Sueada* (62.4%); south arm *Salicornia* (57.3%), *Sueada* (42.9%)] (**Figure 2**). *Nectria* species have been detected in salterns (Chung et al., 2019), in microbial mats in Puerto Rico (Cantrell et al., 2013), and *Nectria punicea* was cultivated from north arm brine (**Table 1**).

The genus *Penicillium* boasts species that are known to mitigate salt and nutrient stress in plants in extreme environments (e.g. Molina-Montenegro et al., 2020), and we found *P. chrysogenum* associated with south arm *Suaeda* plant roots (15.4%) (**Figure 2**). Although our eDNA scan did not locate this genus elsewhere, several species were previously cultivated: in the north arm *P. buchwaldii* (brine) and *P. aff. mononematosum* (ooids), and in the south arm *P. crustosum* (brine) (**Table 1**).

*Powellomyces hirtus* was very the most abundant fungus present (84%) in the eDNA study of microbialite structures (**Figure 2**). These mats support pupation of the *Ephydra* flies, and in the pupae themselves, *P. hirtus* was also the most abundant (91%). *Powellomyces* is considered a soil-dwelling taxa, and it has also been observed growing inside the protective casing of pollen grains (Longcore et al., 1995). This species may be considered parasitic as it appears to negatively impact plant growth (Wang et al., 2021). The abundance in the mats and pupae suggest *P. hirtus* may be thriving in the south arm lake ecosystem, but it’s role there is unclear.

The genus *Rhizophlyctis* includes 36 species of ubiquitous soil-inhabiting chytrids (Letcher et al., 2008). *Rhizophlyctis rosea* was identified from north arm soil (6.9%), as well as in the rhizosphere of Suaeda plants (4.7%) (**Figure 2**). *Rhizophlyctis rosea* has been shown to survive in dry soil for extended periods, then resumes growth after rainfall or other moisture (Willoughby, 2001). *Rhizophlyctis rosea*, which is one of the most often observed soil chytrids, is a cellulose decomposer (Letcher et al., 2008).

*Wallemia* is a common inhabitant in hypersaline environments, and thus it was anticipated that this genus might be at Great Salt Lake. Indeed, *W. muriae* was cultivated from north arm brine and *W. sebi* from south arm lake water (**Table 1**). *W. sebi* DNA *was also* amplified at the north shore tar seeps (3.3%) (**Figure 2**). Taxonomically, *W. sebi* is a complex of at least four species and among the most xerophilic eukaryotic life (Jančič et al., 2015). *Wallemia* have been isolated from dust and other low water activity (a_w_) habitats (Zalar et al., 2005), and its spores are known to be air-disseminated (Zeng et al., 2004).

## 5 Roles for fungi at Great Salt Lake

### 5.1 Alleviation of salinity stress

The soils surrounding Great Salt Lake are impacted by two major stressors of plant growth: lack of fresh water and high soil salinity (Bradbury and Parrott, 2020). While fungi cannot increase the availability of water in the soil, they have been shown to mitigate plant salt stress by forming symbiotic relationships with plants, either in the rhizosphere or as endophytes (Gupta et al., 2021). Several genera found in our eDNA including *Penicillium, Alternaria*, *Aspergillus*, and *Trichoderma* appear to reduce salt stress-induced pathways in plants by producing osmoprotectants to prevent osmotic stress (Tiwari et al., 2023) and can mitigate salt/nutrient stress in plants phosphate-solubilizing (Molina-Montenegro et al., 2020).

Many *Ascomycota* genera, including *Aspergillus*, can improve soil fertility, regulate water uptake and distribution, and produce compounds to regulate ion uptake and transport in plants (Aizaz et al., 2023; Lubna et al., 2022; Abeer et al., 2014). *Aspergillus eurpaeus* and *Fusarium* sp. accumulate solutes such as polyols and free amino acids under salt stress to change their osmotic potential and allow water to enter their cells (Chamekh et al., 2019). Fungi have evolved other strategies which allow for growth in hypersaline conditions, such as changes in plasma membrane fluidity (Gunde-Cimerman et al., 2009). In plant root microbiomes, for example *Penicillium chrysogenum* found in *Suaeda* roots, the presence of such microorganisms makes sense to mitigate the salt encountered in the soils. But other species associated with the Great Salt Lake microbialites present novel concepts of symbiosis in mat communities. *Acaulospora kentinensis* represents 55% or greater of the fungal abundance in the microbial mats. This genus is associated with the roots of Asian grasses where increases salt tolerance for the plants (Wu et al., 1995). Could this strain alleviate salt stress for a microbialite consortia? *Diversispora eburnea,* an arbuscular mycorrhizal fungus in the lake mats, might also work in this regard for the microbialites; a study previously showed that this species improved maize growth under saline stress (Sun et al., 2022).

### 5.2 Nutrient cycling

One of the roles of fungi in ecosystems is decomposition, facilitating the cycling of various nutrients. While saline or hypersaline environments can negatively impact fungal diversity (Saifullah Dahlawi et al., 2018), we have identified several in our dataset which are involved in nutrient cycling including *Fusarium*, *Trichoderma*, *Penicillium*, *Alternaria*, *Cladosporium*, *Aspergillus* and *Coniochaeta* (Egidi et al., 2019). The genus *Aspergillus* (north arm tar seeps) has been shown to solubilize insoluble phosphates, such as Ca, Fe and Al phosphate, and to recycle carbon and nitrogen through the environment (Nayak et al., 2020). It has also been found in biocrusts in southern Utah, where it plays a role in soil stability and plant biomass decomposition (Challacombe et al., 2019). *Coniochaeta* species (north arm water and tar seeps) have also been implicated in plant biomass decomposition and carbon and nitrogen cycling in arid ecosystems (Challacombe et al., 2019), as have *Penicillium* species (south arm *Suaeda* soil samples) (Kuypers et al., 2018).

### 5.3 Decontamination and remediation

Hydrocarbons are a rich source of energy for fungi and other microorganisms, some of which can use hydrocarbons as a sole carbon source (Prince and Walters, 2016). The Great Salt Lake microbial community would certainly utilize the rich petroleum carbon source in this extreme environment (Prince and Prince, 2022). At Great Salt Lake, several species of fungi were identified that may have the ability to degrade the polycyclic aromatic hydrocarbons (PAH) that characterize the Rozel tar seeps (Damsté et al., 1987; Prince, 2019). *Cladosporium cladosporioides* (Bakri, 2022; Birolli et al., 2018), *Penicillium citrinum* (Birolli et al., 2018), *Penicillium commune* (Esmaeili and Sadeghi, 2014), and *Epicoccum nigrum* (Queissada et al., 2013). *Rhodotorula marina* was identified at the Rozel seeps as well and some species of marine Rhodotorula have been found to degrade PAH’s (Shailubhai et al., 1984; Lin et al., 2022) so it is possible that this Great Salt Lake species also has this capability. The most abundant fungal species identified at the seeps was *Aspergillus niger*, a ubiquitous fungal species that is present in other petroleum-rich sites and degrades hydrocarbons including high molecular weight tar (Zafra et al., 2014; Mauti et al., 2016; Barnes et al., 2018; Nayak et al., 2020). Polluted soil was used to inoculate enrichment cultures to observe the microbiota that degrade PAHs; the enriched fungi composition included *Aspergillis* (Spini et al., 2018). The genus, *Fusarium*, was the most abundant fungal taxa, and we discovered a species in gypsum crystals near the Rozel tar seeps. Also, Trichoderma was prevalent in their data, and we located it in the north arm brine column.

Heavy metals in soils may also be mitigated by some species of *Aspergillu*s (e.g. Ai-Jawhari, 2014; Nayak et al., 2020), which is a significant attribute at Great Salt Lake, a terminal basin where metals have accumulated due to natural and mining sources (e.g. Naftz et al., 2008; Adams et al., 2015; Wurtsbaugh et al., 2020). In the microbialites, the arbuscular mycorrhizal fungus, *Diversispora eburnea*, aids plants in dealing with heavy metals (e.g. Buck et al., 2019, Faggioli et al., 2019; Sun et al., 2023), and it begs the question if it may have a similar function in microbial mats of Great Salt Lake where metal contamination has become an environmental concern (Adams et al., 2015).

### 5.4 Plant growth promotion

The shoreline soils surrounding Great Salt Lake are home to many halophytic plants, including *Distichlis spicata*, *Salicornia utahensis*, *Suaeda erecta* and others (Bradbury and Parrott, 2020). These plants are adapted to thrive in this high salt, low water environment, but recent research has pointed to halophilic fungi inhabiting the plant’s rhizosphere may play an outsized role both germination and growth. Several *Ascomycota* genera have been shown to promote seed germination and promote plant growth by altering rhizosphere hormone balance thus changing phytohormone plant signaling pathways (Dodd et al., 2010; Verbon and Liberman, 2016). *Aspergillus*, *Trichoderma* and *Microascus* have all been found in various locations around the lake (**Figure 1**) and are implicated in bio-priming of seeds (Aizaz et al., 2023). *Cladosporium* species*,* found in the rhizosphere of both *Salicornia* and *Sueda* in the south arm of the lake, have been previously implicated in the production of protein hydrolysates (PHs), a class of biostimulants, which act as a plant growth-promoting agent, protecting plants from biotic and abiotic stressors (Răut et al., 2021).

### 5.5 Desiccation resistance

One survival strategy in terminal lakes, which vacillate in elevation, is resistance to desiccation during periods of drought or seasonally high rates of evaporation. Many of the fungi of saline systems are adept at surviving periods of dryness, for example, the genus *Wallemia* is observed in low water activity (a_w_) habitats such as salterns or dust particles (Zalar et al., 2005). But some fungi may enable desiccation resistance to the larger community. *Mingxiaea siamensis*, formerly *Bullera siamensis* (Wang et al., 2011), was abundant in the microbialite mats of Great Salt Lake (**Figure 2**). This genus of yeast is associated with biofilm production in marine and saltern habitats (Zajc et al., 2017.), which can aid in combatting the dehydration associated with those environments. Microbialite mats must resist cycles of desiccation, especially in drought years, and *M. siamensis* could play a crucial role.

### 5.6 Virulence and predation

Predator prey relationships are important in maintaining balance in any ecosystem, but in extreme environments, the predators are often microbial. In the brine fly pupae, a species matching *Powellomyces hirtus* was the most abundant fungus (about half of all amplicons), and it also is part of the fungal consortia of the microbialites (**Figure 2**), where the fly larvae dine on microbial mats and pupate. These chytrids are known to predate algae and have been found associated with diatoms (Kilias et al., 2020) such as the *Navicula* species found in the mats (Lindsay et al., 2017). Chytrids are also predators for cyanobacteria, which are also important in microbialite mat formation (Gleason et al., 2015) Although *Coniochaeta* and *Aspergillus* species, such as those found in the Rozel Tar Seeps (**Figure 2**), have been implicated in virulence in Utah desert plants, these taxa are not likely virulent to the lake biology. Instead, they more probably were introduced by airborne plant material or spores (Challacombe et al., 2019).

## 6 Insights and conclusions

Given the paucity of data on fungi at Great Salt Lake, our survey began with the spatial approach of “who” is where. This is challenging considering the nuances of an ever-changing Great Salt Lake, thus it is critical to understand the study site. In interpreting the eDNA data, we encountered sequences that were likely not part of the lake system but may have introduced from nearby farm fields or grasslands. Case in point, *Ustilago bullata*, an infectious agent causing smut in corn and grasses, was implicated in soil and shoreline plant roots (**Figure 2**), but it is not known to be associated with these types of halophytes. Also, the mycobiome of Great Salt Lake microniches may differ from similar habitats elsewhere; for example, European *Salicornia* root fungi communities are distinct (Furtado et al., 2019).

The diversity of soil types at Great Salt Lake (Bradbury and Parrott, 2020) alone presents a broad range of fungal habitats. Oolitic sand, formed in the lake water from the precipitation of calcium carbonate around organic pellets in concentric layers, surrounds the lake (Mathews, 1930). Clay forms where groundwater seeps around dried polygons of oolitic playa (Beaume et al., 2024), and gypsum crystals precipitate here (Eardley and Stringham, 1952). The microbiota of these varied soils are likely distinct based on particle size, geochemistry, water interactions, and the presence or absence of plants (Bradbury and Parrott, 2020).

Another factor that creates variability is water level. As a terminal lake, Great Salt Lake varies in elevation depending on how water inputs for any given year balance with evaporation rates. Some of the spaces analyzed in this study that are now sandy plant habitat could have been under water and devoid of vegetation a few years ago. Whereas this spatial approach gives us important survey data, a longitudinal temporal approach would be necessary to capture the mycobiota of this changing ecosystem over time.

## Data Availability

The data presented in the study are deposited in the NCBI GenBank Sequence Read Archive (SRA) database repository, accession numbers PRJNA847570, PRJNA1131005, and PRJNA1147725.
